# What to Expect from COVID-19 and from COVID-19 Vaccine for Expecting or Lactating Women

**DOI:** 10.3390/pediatric14020034

**Published:** 2022-05-30

**Authors:** Roberta Gangi, Angelica Corrias, Roberta Pintus, Maria Antonietta Marcialis, Vassilios Fanos

**Affiliations:** 1 School of Pediatrics, University of Cagliari, 09124 Cagliari, Italy; roberta.gangi@hotmail.it (R.G.); angelicacorrias03@gmail.com (A.C.); 2 Department of Surgery, University of Cagliari and Neonatal Intensive Care Unit, AOU di Cagliari, 09124 Cagliari, Italy; ma.marcialis@libero.it (M.A.M.); vafanos@tiscali.it (V.F.)

**Keywords:** SARS-CoV-2, COVID-19 during pregnancy, pregnancy, lactating, vaccination, vaccine hesitancy, mRNA vaccines, passive immunization

## Abstract

Recent studies identified pregnancy as a high-risk condition for the development of maternal-fetal complications in the case of the SARS-CoV-2 infection. Therefore, the scientific community is now considering pregnant women a “fragile” category that should be vaccinated with high priority. The number of pregnant women undergoing hospitalization since summer 2021, including Intensive Care Unit admission, is growing, as well as the risk of preterm birth. Evidence from both animals and humans suggest that, similarly to other vaccines routinely administered in pregnancy, COVID-19 vaccines are not crossing the placenta, do not increase the risk of miscarriage, preterm birth, stillbirth, the birth of small gestational age neonates, as well as the risk of congenital abnormalities. To date, the World Health Organization and scientific literature are promoting and encouraging the vaccination of all pregnant and lactating women. The aim of our narrative review is to present the available literature regarding this issue with the aim to provide appropriate answers to the most frequent requests, doubts, and fears that have led many expecting and lactating women not to become vaccinated during this pandemic period.

## 1. Introduction

At the current time, the COVID-19 pandemic has caused 5,195,138 deaths around the world and the total number of infected people since March 2020 amounts to 261,075,046 (data updated to 28 November 2021 h10:16). As it emerged from previous SARS (2022), MERS (2009) and H1N1 (2012) outbreaks [1,2,3,4,5], pregnant women have stood out for being subjected to more severe outcomes compared to the general population [6,7,8,9,10,11]. A study has shown that the mortality risk in pregnant women with COVID-19 is 22 times greater than that in non-infected pregnant women [12]. The more severe course of SARS-CoV-2 infection during pregnancy has led to a three-fold increase in preterm births, as interestingly emerged from a meta-analysis [13]. Anti-COVID-19 vaccination is therefore a priority vehicle to reach both maternal and neonatal/fetal protection. The non-inclusion of pregnant and lactating women in clinical trials of SARS-CoV-2 vaccines and the subsequent lack of reliable data about their safety and efficacy in this specific group of people, have been responsible for the creation of a climate of mistrust and “vaccine hesitancy”. Many women have decided to refuse or delay the vaccination, scared by the possibility that this could represent a threat to fertility, to the product of conception, or to maternal milk properties. Many health care professionals—not adequately informed about anti-SARS-CoV-2 vaccines’ characteristics and mechanism of action—have adopted a skeptical position, reinforcing resistant and oppositive behaviors of a multitude of women against the vaccination. It is therefore necessary and urgent to convey correct information about this topic, based on scientific evidence obtained to date, to women that are going through this stage of life and to their health professional referrals. Our article covers a range of issues within this topic, but we did not follow rigid selection criteria since it is a narrative review. Specific selection criteria are proper for systematic reviews. We searched different databases, including PubMed and Google scholar, in order to identify the majority of relevant studies, almost one hundred in total.

**Figure 1:**
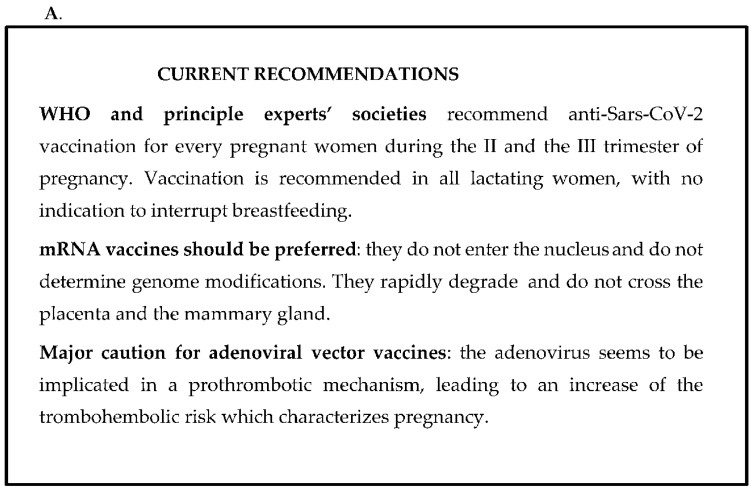

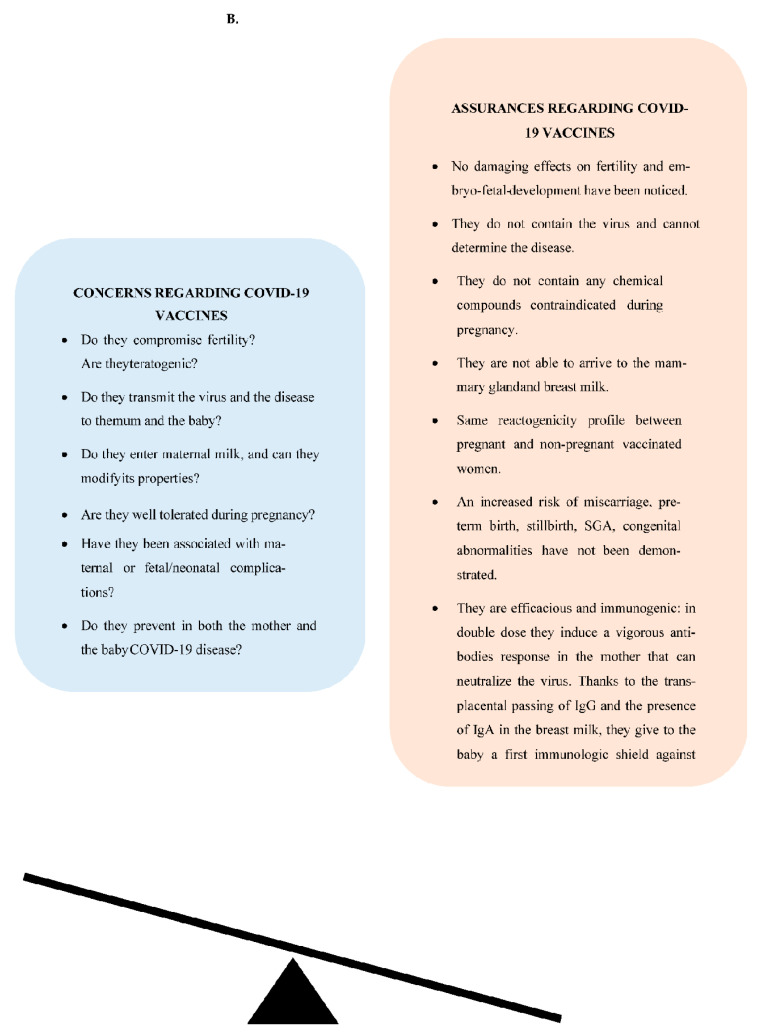
(**A**,**B**). The current WHO recommendations (**A**) and the main concerns and assurances regarding the anti-COVID-19 vaccination (**B**).

The current WHO recommendations and the main concerns and assurances regarding anti-COVID-19 vaccination are summarized in Figure 1A,B.

## 2. COVID-19 during Pregnancy

### 2.1. What Is the Impact of COVID-19 on Pregnant Women?

Several studies demonstrated that pregnancy is an additional risk factor for a more severe course of SARS-CoV-2 infection [6,7,8]. Currently available data suggest that pregnant women with symptomatic infection develop a more severe disease compared to non-pregnant women [9,10,11]. This leads to an increase in terms of Intensive Care Unit (ICU) admission, need for mechanical ventilation, and for cardiocirculatory and pulmonary support through Extra-Corporeal Membrane Oxygenation (ECMO) [11,12,13,14,15,16]. A multicentric observational study performed on 2130 pregnant women (796 with SARS-CoV-2 infection and 1424 not infected), shows that the risk of maternal mortality is 22 times higher in the COVID-19 group compared to the control group of non-infected women [12]. The percentage of ICU admission increases with increasing gestational age. In fact, an article reported that the more than 90% of pregnant women involved in the study needing ICU admission were in the third trimester of pregnancy [17]. Moreover, in addition to the appearance and circulation of new SARS-CoV-2 variants, a higher maternal morbidity has been observed with the pandemic progression. Indeed, a prospective cohort Italian study, performed between February 2020 and June 2021, evidenced a three-times higher risk of the need for mechanical support and/or ICU admission in pregnant women (odds ratio adjusted for age, citizenship, previous comorbidities, and obesity is 3.04; IC 95%: 1.72–5.37) [18]. It is believed that this increase is due to the Alpha variant of the virus, which has spread more and more since the first trimester of the study and is probably associated with major maternal morbidity [19]. A recent analysis of English data (updated to 11 July 2021) underlined a major risk of maternal and perinatal morbidity. This could also be associated with the Delta variant circulation, which has occurred since mid-May 2021 [20].

### 2.2. What Are the COVID-19 Symptoms in Pregnant Women?

Three-quarters of pregnant women with SARS-CoV-2 infection are asymptomatic, similar to what has already been observed in non-pregnant women [21,22]. According to a metanalysis, pregnant women with COVID-19 develop symptoms less frequently compared to non-pregnant women [13]. When in its symptomatic form, the SARS-CoV-2 infection manifests itself with moderate signs such as cough (41%), fever (40%), dyspnoea (21%), and myalgia (19%) during pregnancy [23]. Lymphopenia and increase of C-reactive Protein (CRP) are the laboratory parameters’ modifications most frequently found [13]. Cough, chest pain, and dyspnoea have shown a considerably higher incidence among hospitalized pregnant women. This is probably related to a more severe infection and therefore cardio-pulmonary symptoms are the discriminating factors for hospitalization [23]. Furthermore, it has been noticed that fever and dyspnoea are associated with a higher probability of maternal complications [12].

### 2.3. Are There Factors Impairing COVID-19 Course in Pregnancy?

The higher susceptibility to the development of complications in case of SARS-CoV-2 infection among expectant mothers could be partly due to physiologic changes that occur during pregnancy. The stability of the cardio-pulmonary system during the infection may become precarious due to the following aspects: the increase of cardiac frequency, the subsequent oxygen consumption, the progressive reduction of total pulmonary capacity with the increase of uterus encumbrance (which leads to diaphragm elevation) [12]. Moreover, the inflammatory hyperactivity, the endothelial dysfunction, and the subsequent platelet activation, which are typical aspects of the infection, add up to the well-known pro-thrombotic and procoagulant modifications that occur during pregnancy. This enhances the risk of thromboembolic events, that can also affect the placental circulation and undermine its integrity and function, leading to an increased risk of pre-eclampsia, Intrauterine Growth Restriction (IUGR) and preterm birth [24]. In this regard, it has been highlighted that COVID-19 during pregnancy is associated with an increase in the risk of venous thromboembolism from 0.2 to 6%, with higher D-dimer levels compared to non-infected expectant women [21,25]. There is one more factor that could contribute to the severity of SARS-CoV-2 infection in pregnant women: the immunological system modulation in the maternal organism, which is necessary to create tolerance towards the product of conception so as to prevent the rejection. This could make the immunological response to the virus suboptimal. During pregnancy, the hormonal influence, especially oestrogens and cortisol, promotes a shift in anti-inflammatory immunological activity. The consequences are a minor production of pro-inflammatory cytokines and a decrease in T-Helper type 1 lymphocytes in favour of type 2 [26,27]. This could explain the reason pregnant women often have a more severe clinical progression during viral infections (e.g., influenza, HEV, HSV and others).

### 2.4. Which Maternal Comorbidities Can Impair COVID-19 Course in Pregnancy?

During pregnancy, there are some comorbidities that can worsen the COVID-19 disease course, similar to what has been observed in the general population. These comorbidities are: BMI > 30, diabetes mellitus, gestational diabetes, pre-gravid chronic hypertension, preeclampsia, and asthma [13]. Furthermore, there are some risk factors that can worsen the course of this pathology as well, such as maternal age > 35. Moreover, in Asian and black populations the course of COVID-19 seems to be more severe compared to other ethnicities [13]. Indeed, in a study considering a group of pregnant women with COVID-19 in critical conditions that required intensive care admission, 60% of them had gestational diabetes and BMI > 25 kg/m^2^ [28]. Furthermore, it has been reported that the most frequent conditions among pregnant women hospitalized for COVID-19 severe disease, were pre-gravid BMI ≥ 30 kg/m^2^ (41.7%) and type 2 diabetes mellitus (12.5%). Women who died because of COVID-19 (12.5%) all had the same characteristic: they were obese before pregnancy [16].

Speaking about COVID-19 vaccination, in order to correctly evaluate the risk-benefit assessment, women with this condition should consider that the probability of developing a severe form, with a possible impact on the fetus and on the neonate, is significantly high.

### 2.5. What Is the Impact of SARS-CoV-2 Infection during Pregnancy on the Fetus and Newborn?

There is no clear evidence that SARS-CoV-2 infection during the first trimester of pregnancy could increase the risk of early miscarriage [29]. Although this is encouraging data, information regarding the early stages of pregnancy is limited and they mostly regard asymptomatic, non-hospitalized women who probably developed a less severe form of infection. On the contrary, some studies demonstrate an increase in preterm births (<37 gestational weeks) in women infected by SARS-CoV-2 compared to non-infected women [13,16,30,31].

A three-time superior incidence of preterm birth is what emerged from a metanalysis [13]. Authors believe that this increase could be also related to the decision made by the obstetric staff to induce labor before the traditional term, in order to guarantee the best ventilatory assistance to mothers with severe COVID-19. Similarly, an Italian prospective cohort study showed that 10.9% of pregnancies ended up with preterm labor, exceeding the pre-pandemic national average of 6.7% [18]. Another article demonstrated a two-fold increase regarding fetal distress and a three-fold rise in terms of perinatal/neonatal mortality and morbidity risk among children of mothers with SARS-CoV-2 infection [12]. This increase in fetal and perinatal mortality could be justified, in some authors’ opinion, by the occurrence of microthrombi in the placental circulation and by the subsequent reduction of placental perfusion in pregnant women with COVID-19 [32].

### 2.6. Is There the Risk of Transmitting the SARS-CoV-2 Virus to the Fetus/Newborn?

It has been noticed that the risk of transplacental transmission is low, around 3% [33], and it is not associated with the development of severe comorbidities in neonates [34,35,36,37].

The transmission is very rare, and it could be explained by the minimal expression of angiotensin-converting enzyme 2 receptor (ACE-2) and Transmembrane protease serine 2 (TMPRSS2) by the placenta [38,39].

In a study conducted on 261 neonates of mothers infected by SARS-CoV-2, 10% of the performed molecular virus detection tests using nasal swab had a positive result, and 20% of them presented with fever or mild dyspnea, without any serious complications. C-Reactive Protein (CRP) analyses on umbilical cord blood, placenta, and vaginal secretions did not find the virus. This suggests that the transmission is likely to be postnatal more than transplacental [37]. Regarding the possibility of virus transmission through breast milk, it is unlikely to be possible. In a study involving 64 infected mothers, only one milk sample tested positive for SARS-CoV-2 RNA research. However, no replicating virus was found [40,41].

## 3. COVID-19 Vaccination

### 3.1. Does Vaccination Represent a Novel Concept during Pregnancy?

No, it does not. Thirty years after the development of the first influenza vaccine in 1930, an inactivated monovalent preparation used in the United States military [42], the United States public health authorities recommended the vaccination of pregnant women. In 1997, this recommendation was endorsed. In 2009, Australia and the United Kingdom both recommended vaccines for pregnant women [43]. Unfortunately, despite its proven safety and efficacy, vaccination rates in pregnant women remain low (50% in US and 45% in UK). Other examples of vaccines that can be successfully administered to pregnant women are smallpox, tetanus, and pertussis vaccinations [44].

### 3.2. What Is mRNA Vaccines’ Story?

The story starts in 1987, when Robert Malone mixed strands of messenger RNA with droplets of fat that were absorbed into human cells and started to produce proteins from it [45]. Malone immediately realized that mRNA could be used as a vaccine. In the beginning, mRNA was considered excessively unstable and expensive. However, following some innovations, including chemically modified RNA, it become suitable [46]. The first in vivo study on animal models involved the mRNA vaccine against the Influenza A virus. Subsequently, it has been also used against HIV1, Zika Virus, and rabies virus [47,48]. Moderna and Pfizer BioNTech vaccines use chemically modified mRNA to replace the uridine with pseudo-uridine, to stop the immune system reaction against mRNA, surrounded by carrier bubbles of fats. The mRNA promotes spike protein production [46].

The massive deployment of scientific and financial resources against the COVID-19 pandemic has represented the final push to the maturation and affirmation of this vaccination technology. Pfizer-BioNTech was the first mRNA vaccine that exceeded clinical trials and was the first to be approved for human administration. mRNA can be easily produced, and this makes its creation economic and widely available.

### 3.3. An Old Yet New Concept, Protein Based Vaccine against SARS-CoV-2, the Future Is Near?

There are several protein-based vaccines against other pathogens available, but on December 2021, the Technical Advisory Group for Emergency Use Listing listed Nuvaxovid (NVX-CoV2373) and Covavax vaccine against COVID-19 for emergency use [49]. It is the first protein-based COVID-19 vaccine to be used in Europe, also called Novavax. It was created by recombinant protein technique. It contains the spike proteins (antigen) and an adjuvant (a saponin). This adjuvant generates a strong immune response and allows to lower the dose of spike protein itself in the preparation. This vaccine was made by incorporating the spike genes into a baculovirus that infected moth cells. The spike proteins produced are then purified and assembled into nanoparticles. This technology is one of the first steps to create a universal vaccine design platform (to be used to create vaccines against any pathogen), even if some authors proposed to engineer bacteriophage T4 [50,51]. There are no immunogenicity or safety data concerning the administration of Novavax in pregnant and breastfeeding women. Nevertheless, animal studies have not shown any direct or indirect damage related to pregnancy or embryonic/fetal development [52]. Furthermore, based on previous evidence from other protein-based vaccines during pregnancy, efficacy is expected to be comparable to non-pregnant women of a similar age [49].

### 3.4. Should Anti COVID-19 Vaccination Be a Priority among Pregnant Women?

Since several studies identified pregnancy as a dangerous condition in the case of the SARS-CoV-2 infection, in terms of both maternal and fetal complications, COVID-19 vaccination among pregnant women is a priority prevention tool [13,14]. Vaccination would create an immune shield not only for the mother, but also for the newborn, due to the antibodies’ capacity to cross the placenta. Thanks to this, the newborn can benefit from the passing of maternal antibodies for the period immediately following the birth and during the first months of life. During this time, newborns are not only too young to be vaccinated, but also their immune system is still immature and not perfectly performing. The immunologic potential of vaccination during pregnancy is well-known and has been exploited for a long time. It is the case of vaccinations that are routinely recommended in Italy for all pregnant women, particularly the vaccinations against diphtheria-tetanus-pertussis and influenza virus.

### 3.5. What Is the Number Needed to Vaccinate to Avoid Harm (NNV) for COVID-19 Vaccination?

Generally speaking, COVID-19 vaccination in pregnant women should be considered after an estimation of the vaccine’s risks and benefits. To do this, Magee LA et al. considered the Number Needed to Vaccinate (NNV). The authors claim that vaccination is recommended only if the NNV to avoid maternal or fetal damage is lower than the NNV needed to cause harm. The authors reported an NNV of 11 to prevent infection, an NNV of 206 to prevent a symptomatic form of infection, an NNV varying between 412 and 2058 to prevent severe disease, and an NNV ranging from 1371 to 6857 to avoid a case needing mechanical ventilation. They also indicated that adverse post-vaccination events are rare, as demonstrated by the fact that the NNV necessary to cause damage is 37,000 for myocarditis and 50,000 for thrombotic thrombocytopenic syndrome [53].

### 3.6. Should All Pregnant and Lactating Women Have Been Included in COVID-19 Vaccine Development from the Outset?

Pregnant and lactating women have not been included in phases II and III of clinical trials for COVID-19 vaccines. The start of the vaccination campaign occurred in a climate of disorientation and uncertainty of these categories of subjects.

As a result of the lack of conclusive data regarding the efficacy and safety of vaccines, many health care professionals discourage the vaccination, and many women refuse it or delay it, scared by the possibility of damaging the fetus and of modifying breast milk properties.

### 3.7. Misinformation about the Impact of COVID-19 Vaccines on Pregnant People: Let’s Be Clear!

It is essential to underline that none of the four authorized vaccines in Europe in order to combat the COVID-19 pandemic (respectively two mRNA and two adenoviral vector vaccines) contain the active virus, and therefore they cannot determine the disease, neither in the mother, nor in the baby. These vaccines cannot lead to DNA alterations, and they do not induce genome mutations. mRNA contained in the Pfizer-BioNTech and Moderna vaccines, like all mRNA produced by cells, naturally degrades in a few days after inoculation and hence it is biologically implausible that it can reach the placenta and the fetus through blood circulation [54,55].

Furthermore, vaccines do not contain any chemical compounds contraindicated in pregnancy and during the lactating period. Obviously, it is different in case of specific allergies to excipients such as polyethylene glycol (contained in Pfizer-BioNTech: BioNTech Manufacturing GmbH, Mainz, Germany; and Moderna: MODERNA BIOTECH SPAIN, Madrid, Spain) and polysorbates (contained in Janssen: Janssen Biologics B.V. Leiden, Netherlands; and Vaxzevria: Henogen S.A., Seneffe, Belgium).

### 3.8. Which Is the COVID-19 Vaccine Currently Authorized for Pregnant Women?

At the moment, there is no absolute contraindication, neither in pregnant, nor in lactating women, for the administration of the four authorized vaccines by FDA and EMA. Regarding adverse effects and maternal, fetal and neonatal outcomes, mRNA vaccines (Pfizer-BioNTech and Moderna) show a larger amount and more reassuring data compared to adenoviral vector vaccines. Data regarding the use of Vaxzevria and Janssen in pregnant women are very limited, even though preliminary animal studies did not show any damaging effect during pregnancy. The recent emergence of thromboembolic events (cerebral venous sinus and splanchnic district thrombosis) after the administration of adenoviral vector vaccines, even though rare (incidence of 4.1/1,000,000 vs. 39/1,000,000 in patients with COVID-19) [56], does impose greater caution in the evaluation of these vaccines during pregnancy, since this condition has itself an increased risk of coagulopathies.

### 3.9. What Are the Current Recommendations?

Today, the WHO and the principal scientific organs promote and encourage vaccination in all pregnant and lactating women. In Italy, on 24 September 2021, the Minister of Health came out in favor of vaccination in these groups of women. They can choose whether to vaccinate or not after a meeting with a health care professional, in order to inform them of scientific evidence on the benefits of vaccination and the potential risks in the case of infection for the mother and the neonate. It was seen that an adequately informative meeting increased women’s compliance from 5 to 50 times [57,58]. Women with a high exposure risk (e.g., health care professionals or caregivers) and/or with health conditions that implicate a major risk for both the mother and the fetus/newborn in case of infection, should be vaccinated with the highest priority.

### 3.10. When Should Pregnant Women Be Vaccinated?

It has already been seen for other vaccines that the transfer of maternal antibodies is maximum during the third trimester of pregnancy [55]. For this reason, experts suggest administering the vaccine between the end of the second and the beginning of the third trimester, so as to guarantee an adequate passage of antibodies from the maternal side to the fetal side of the placenta [59,60]. Moreover, the third trimester is characterized by a higher probability of COVID-19 complications in pregnant women [25]. Regarding the first trimester, no conclusive data about the safety and the potential teratogenic effects have been published, also considering the small number of women vaccinated in this stage. Different observational studies and a review (2020) describe the risk of malformations associated with maternal cold in the first trimester and a significant increase of neural tube defects (OR = 1.92 IC95% 1.61–2.29) [55,61]. Based on these concepts, it should be more advisable to avoid the vaccination during the first trimester. If a vaccinated woman discovers to be pregnant just after the vaccination, there is no evidence that the pregnancy should be interrupted. If this discovery happens between the first and the second dose, the woman could delay the new administration after the birth, except for high-risk subjects.

### 3.11. Is the COVID-19 Vaccine Well Tolerated in Pregnant Women?

Data regarding mRNA vaccines in terms of security and adverse reactions are reassuring. A study conducted on more than 35,000 pregnant women demonstrated a similar reactogenicity profile in pregnant women vaccinated with Pfizer-BioNTech and Moderna (two doses) on one hand, and non-pregnant women on the other hand. Some minor differences have been noticed: injection-site pain was more frequently signalled among pregnant women, while, on the contrary, headache, muscular pain, cold and chills were less often signalled in this group [62].

### 3.12. What Are the COVID-19 Vaccine Related Concerns during Pregnancy? Are the Mothers Afraid of Side Effects for Fertility and for the Fetus?

Developmental and Reproductive Toxicology (DART) pre-clinical studies conducted on animal models did not show any direct or indirect damaging effect of the COVID-19 vaccine regarding fertility, pregnancy course, embryo-fetal development, birth, or postnatal growth. During the trials for Pfizer-BioNTech and Moderna vaccines, respectively 23 and 13 women became pregnant after enrollment and they were followed during pregnancy, without noticing any adverse effects for the mothers or for the fetuses. The number of pregnant vaccinated women on a global level has exceeded hundreds of thousands and no more side effects have been registered compared to the non-pregnant population [63,64].

A study on the safety profile of mRNA vaccines followed 827 pregnant women until the end of pregnancy. Among them, 712 (86.1%) gave birth to a healthy child. Almost all of them received the vaccine in the third trimester of pregnancy. A total of 115 (13.9%) women had a miscarriage, mostly during the first trimester. The same study claimed that this result is comparable to pre-pandemic data, when miscarriages during the first trimester occurred in a percentage varying from 10% to 26% of pregnancies. Other outcomes were represented by one case of stillbirth (0.1%) and 10 cases (1.2%) of voluntary termination of pregnancy and ectopic pregnancies. Premature births have been registered in 9.4% of cases. 3.2% of newborns were Small for Gestational Age (SGA) and 2.2% had congenital defects. Once again, data confirmed the pre-pandemic situation. Among women who gave birth to children with congenital anomalies, none had the vaccine administered during the first trimester or during the pre-conception period and no specific pattern of congenital anomalies has been determined [62].

Additionally, a recent study confirmed that the administration of an mRNA vaccine during pregnancy does not increase the risk of recurrent miscarriage compared to that of the general population (adjusted OR 1.02; 95% CI, 0.96–1.08) [65].

### 3.13. What Is the Effect of COVID-19 Vaccine on the Fetus?

Usually, vaccine administration during pregnancy is immunogenic [66]. Also, for COVID-19 there is strong evidence regarding the transmission of maternal antibodies against SARS-CoV-2 to the fetus after vaccination [67,68,69,70,71]. A study that evaluated 131 fertile women who got vaccinated with Pfizer-BioNTech or Moderna showed in all of them a high quantity of anti-SARS-CoV-2 antibodies (IgG, IgM, and IgA) in the blood after vaccination, with a similar title between pregnant and non-pregnant women. The level of produced antibodies after vaccination demonstrated to be significantly higher (*p* < 0.00001) than the level found in 37 pregnant women evaluated 4–12 weeks after SARS-CoV-2 infection [68].

What is even more remarkable is that antibodies induced by vaccination were also found in samples of umbilical cord blood and in breast milk, clearly suggesting their transmission to the newborn from the placenta and by maternal milk. A recent Israelian study has shown that anti-SARS-CoV-2 IgG title in the umbilical cord blood of neonates born from mothers vaccinated with Pfizer-BioNTech (double dose) in the third trimester was significantly higher (*p* < 0.05) compared to the antibodies title in children of mothers who developed the infection during pregnancy. In the authors’ opinion, the higher the quantity of antibodies produced and transferred from the mother to the fetus, the longer the protection for the newborn against the virus in their first months of life will be guaranteed [72].

### 3.14. Should Lactating Women Receive the COVID-19 Vaccine?

At the moment, no certain data regarding safety of COVID-19 vaccines in lactating women are available, since they have not been included in clinical trials. The Pfizer-BioNTech and Moderna pharmaceutical companies state that mRNA vaccines cannot reach maternal milk: the fragile mRNA, once used, is rapidly degraded. Thus, it is very improbable that it can spread from the cells where it is injected to blood circulation, and it is even less probable that it can arrive to the mammary gland. A study did not find any traces of mRNA after vaccination considering 13 human milk samples taken from 7 women during the 48 h following the vaccine administration (5 Pfizer-BioNTech and 2 Moderna). Authors believe that vaccine’s mRNA, even considering the possibility of its presence in the human milk, would rapidly be destroyed by the stomach’s acid of the neonate [73]. Although this data is based on a limited number of samples, it remains reassuring.

### 3.15. Should the COVID-19 Vaccine during Breastfeeding Be Immunogenic for the Baby?

Anti-COVID-19 vaccination during the breastfeeding period would permit the transport of specific IgA antibodies directed against SARS-CoV-2 from the mother to the infant. These antibodies, in addition to those IgG transmitted through the placenta, would allow the newborns to increase their defenses against the virus. Two studies examined maternal milk samples of women who previously received two doses of the Pfizer-BioNTech vaccine. What was discovered is the substantial presence of IgA and IgG against SARS-CoV-2 in the first week after the first injection. [74,75]. Another encouraging discovery is that antibodies found in maternal milk, in both women who got vaccinated and women infected by the virus, demonstrated, in vitro, to have a neutralizing activity against the virus [74,76,77]. It means they are functional antibodies, capable of eliminating the virus.

### 3.16. How Long the Antibodies Received/Transferred by Breast Milk Last?

According to experts, anti-SARS-CoV-2 antibodies received by maternal milk have a localized and temporary activity in the baby. They do not enter the blood circulation, but they colonize the oral cavity, the pharynx, and the gastro-intestinal tract in general, leading to the construction of a defence barrier against the virus entering. Furthermore, antibodies are digested in the infant’s gut in a few hours: it means that their protection is partial and fails with the end of breastfeeding. A continuous restock is then crucial. At the moment we do not know with certainty for how long vaccinated people will continue to produce antibodies, but existing evidence suggests that it could be a long period. A study asserts that antibodies’ production in people who have received the Moderna vaccine lasts for at least six months [78]. It means that children could receive a moderate level of protection from the mother, if she does not interrupt the breastfeeding. However, it is also true that antibodies’ concentration in breast milk decreases with time [79].

## 4. Conclusions

Our review provides scientific evidence and underlines the importance of the COVID-19 vaccine in pregnancy and lactation and its power to protect two lives at once by preventing severe disease in both mothers and newborns.

During the last months, collected data from various case reports, observational studies, and case-control studies conducted on pregnant women and lactating women vaccinated for COVID-19, have consistently demonstrated that vaccination is effective, safe, and immunogenic even in this group of subjects [80,81,82,83,84,85]. The demonstrated capacity of antibodies to be transmitted through the placenta and breast milk after the vaccine permits newborns to have a first and precious immunologic shield against the virus. This makes COVID-19 vaccination an even more fundamental occasion. To date, an intersociety consensus in terms of recommending vaccination in every expecting and/or lactating woman has been reached. [86,87,88]. Counseling with health care professionals is crucial in order to maximize women’s compliance. Pregnant women are fragile and for this reason, they would primarily be addressed for vaccination. In fact, we must remember that, at the moment, it represents the best weapon we have to combat and arrest SARS-CoV-2 spreading.
